# Bank market structure and firm innovation-evidence from China

**DOI:** 10.1371/journal.pone.0296391

**Published:** 2024-03-13

**Authors:** Hua Shang, Huan Liu, Yanlin Xing

**Affiliations:** Research Institute of Economics and Management, Southwestern University of Finance and Economics, Chengdu, China; University of Rome Tor Vergata: Universita degli Studi di Roma Tor Vergata, ITALY

## Abstract

This study explores the impact of bank market structure on firm innovation in China using a two-way fixed effects model. Our analysis is based on a dataset comprising 2,412,316 firm-city level observations. Our findings suggest that there exists a U-shaped relationship between bank market structure and firm innovation in China. Specifically, we identify that the financing channel is a crucial pathway through which bank market structure influences firm innovation. Moreover, our results indicate that this relationship is mainly driven by small firms and innovative firms, and is more pronounced in regions with stronger intellectual property rights protection or greater openness to foreign markets. In sum, this study contributes to the existing literature by advancing our understanding of the impact of bank market structure on innovation in Chinese firms.

## 1. Introduction

Innovation has been widely recognized as a primary driver of economic growth, as first identified by Solow [1]. In academia, there is growing interest in examining how the development of financial markets affects firms’ innovation [2–6]. Given that the structure of the banking market is expected to have a substantial influence on innovators’ access to financial resources and their risk management strategies, a body of recent research has been dedicated to exploring the intricate relationship between bank market structure and firm innovation. However, these investigations have yielded inconsistent results [7–10]. One plausible explanation for this disparity in findings may be that the majority of prior studies have only examined the linear relationship between bank market structure and firm innovation, overlooking the possible existence of a non-linear relationship. Therefore, this study aims to investigate the non-linear relationship between bank market structure and firm innovation, the underlying mechanisms of this influence, and the potential firm and regional heterogeneities.

The impact of bank market structure on firm innovation can be elucidated through three distinct theoretical predictions. One prediction posits that a competitive market structure can act as a catalyst for firms’ innovation output. The traditional Structure-Conduct-Performance (SCP) hypothesis contends that a competitive banking system exhibits greater efficiency compared to a monopoly system. In a competitive banking environment, firms can access more financing [11, 12] and are better poised to invest in innovative projects, consequently fostering innovation output. Conversely, another prediction suggests that a concentrated banking market structure, characterized by lower competitiveness, can also have a positive impact on firms’ innovation output. The Information Asymmetry hypothesis argues that a concentrated banking system proves advantageous for firms with uncertain prospects and opaque information, owing to the presence of information asymmetry between firms and banks. Under such a concentrated structure, banks are more inclined to provide support to firms, as they can establish long-term relationships with them and stabilize interest earnings over the entire duration of projects [13]. Given the uncertain and cumulative nature of payoffs from innovative projects, a more concentrated banking system can be conducive to fostering innovation [14]. From the project selection perspective, Huang and Xu [15] illustrates that a competitive bank structure fosters the advancement of technologically intensive R&D projects, whereas a monopolistic bank structure is inclined to support R&D projects including less technology.

A non-linear relationship between bank market structure and firm innovation could exist due to several reasons. Firstly, a range of diverse theories can account for the relationship between bank market structure and firm innovation. This suggests that both positive and negative effects could exist, thereby a non-linear relationship may exist. Notably, the theory presented by Huang and Xu [15] explicitly demonstrates that both monopoly-type and more competitive bank market structures can enhance firm innovation input, thereby implying the presence of a non-linear relationship between bank market structure and firm innovation. Secondly, mixed empirical evidences point to the existence of both positive and negative effects within the same country, which further implying a non-linear relationship. For instance, Amore et al. and Chava et al. [9, 16] have found that a more competitive bank market structure leads to increased innovation output for listed manufacturing firms, young, and private firms in the United States. In contrast, Cornaggia et al. [10] have shown that a more concentrated bank market structure results in greater state-level innovation outputs and innovation among public firms in the United States. Thirdly, Patti and Dell’Arriccia [14] have pointed out the coexistence of the Structure-Conduct-Performance (SCP) effect and the information asymmetry effect and found a bell-shaped relation between bank market structure and firm creation. This also indicate that a non-linear relation between bank market structure and firm innovation could exist.

China, as one of the world’s largest emerging economies, offers an exceptional case study for our investigation. There are several reasons for this choice. Firstly, a crucial aspect of investigating the relationship between bank market structure and firm innovation is the breadth of the range of bank market structures, encompassing both highly concentrated and highly competitive systems. Chinese data provide a wide spectrum, enabling robust results. Secondly, the Chinese banking market operates in city-specific manner. The branches of banks in one city are discouraged to lend to firms in other cities to reduce overlapping competition. This allows for a more precise identification of the impact of the bank market structure of a city. Thirdly, the bank market structure in each city differs considerably, providing ample variation for estimating the relationship between bank market structure and firm innovation. Fourthly, due to the underdevelopment of other financial institutions in China, non-listed firms mainly rely on bank loans, enabling the avoidance of many other confounding factors. Fifthly, China boasts a vast dataset comprising approximately 70,000 observations annually, covering both non-listed and listed manufacturing firms. This extensive dataset better represents the Chinese manufacturing sector and allows for the reliable examination of the impact of bank market structure on the sector.

Our findings reveal a U-shaped relationship between bank market structure and firm innovation in China. This U-shaped relationship is further validated through the U-shape test proposed by Lind and Mehlum [17], reinforcing the credibility of this relationship. Our results are also robust to instrumental variable regression, two-year-ahead innovation output, other proxy of bank market structure and alternative sample period. Furthermore, we identify that the financing channel is a crucial pathway through which bank market structure influences firm innovation. In addition, we demonstrate that this relationship is mainly driven by small firms and innovative firms, and is more pronounced in regions with stronger intellectual property rights (IPR) protection or greater openness to foreign markets.

This study makes several contributions to the existing literature. Firstly, while the impact of bank market structure on firm innovation output in China has been a subject of study, most prior research has concentrated on the linear effect of bank competition on firm innovation [18, 19]. Thus, we depart from prior studies that focused on the linear relationship between bank market structure and firm innovation by proposing that a U-shaped relationship may exist. Our results confirm that as bank market structure shifts from concentrated to competitive, the innovation of firm first decreases and then increases. Secondly, we extend the literature by providing a detailed explanation of the underlying mechanisms and the features of the firms and cities that mainly drive the U-shaped relation. Thirdly, our research additionally offers insights into the relationship between bank market structure and firm innovation in emerging markets. Given that emerging markets have unique economic and financial characteristics, our findings offer insights into how bank market structure influences innovation in these settings.

Our research is related to but differs from the works of Liu and Li [18], Xia and Liu [20] and Zhang et al. [21]. In contrast to Liu and Li [18], who primarily examines the linear relationship between bank market structure and firm innovation output and only demonstrates a non-linear relationship in their robustness check, our primary focus lies in exploring the non-linear relationship between bank market structure and firm innovation output. Additionally, we explored the underlying mechanisms and the heterogeneous effects associated with the non-linear relationship. Unlike Xia and Liu [20], who investigate the non-linear relationship between bank competition and corporate R&D investment, we center our attention on understanding how bank market structure influences firm innovation output and reach different conclusions. Furthermore, in contrast to Zhang et al. [21], our research advances the further understanding of the non-linear relation between bank market structure and firm innovation. Firstly, we establish that the financing channel is a driving force behind the U-shaped relationship between bank market structure and firm innovation. Secondly, our analysis highlights that small and innovative firms are the primary drivers of this relationship. Lastly, our findings reveal that the relationship is more pronounced in regions with better IPR protection or higher degrees of openness to foreign markets.

The remainder of the paper is structured as follows: Section 2 offers a comprehensive review of the relevant literature. Section 3 outlines the Chinese banking market structure. Section 4 introduces the sample and measures, and provides summary statistics for the variables used in this study. Section 5 presents the results and an analysis of the robustness of the findings. Section 6 provides the underlying mechanisms. Section 7 presents further analysis. Section 8 concludes.

## 2. Literature review

Our research contributes to the literature on the impact of banking market on innovation at the industry or firm level. In 1911, Schumpeter argued that financial systems could enhance innovations. Since then, researchers have attempted to explore how bank market development affects resource allocation, thus influencing firms’ innovation. However, there is still no consensus on how bank market development affect firms’ innovation. Proponents argue that banks, as financial institutions, can promote innovation through more efficient resource allocation [22–24]. Conversely, opponents argue that banks may hinder innovation due to their risk aversion and ability to extract rents from firms through information production [2, 25, 26]. In the empirical analysis, Benfratello et al. [8] find that bank market development in Italy promotes the probability of process innovation. Hsu et al. [2] use cross-country data and demonstrate that credit market development discourages innovation in EFD industries and high-tech industries. Shang et al. [27] reveal that provincial-level credit market development promotes firms’ new product sales in China.

Previous research has mainly relied on aggregate measures to assess bank market development, but these measures often fail to capture the market structure. Therefore, there has been a growing interest in investigating how bank market structure affects innovation, leading researchers to delve deeper into this topic. Several theoretical studies have illuminated the impact of banking market structures on economies, particularly on corporate innovation, with researchers developing models to elucidate the macroeconomic distinctions between economies characterized by competitive and monopolistic banking regimes. Scholars, such as [13, 28–31], have employed partial equilibrium models to confirm that a monopolistic banking market can enhance the lending environment. Moreover, Petersen and Rajan [13] argue that high bank concentration encourages firms to form long-term relationships with banks, which increases the willingness of banks to lend to firms with uncertain prospects or credit constraints. Since innovation projects are in general uncertain, this implies that high bank concentration improves firms’ innovation outputs [32]. Conversely, Smith [33] argues, using a general equilibrium model, that a non-competitive banking market is detrimental to economic growth. Despite monopolistic banks potentially fostering technological innovations and reducing screening costs, these advantages are offset by the redistribution of productive resources in the form of profits to the banks. Guzman [11] argues that monopoly banking system is less efficient than competitive banking system, therefore they cannot provide sufficient funds for innovation. Biswas & Koufopoulos [12] set a model and predicts greater bank competition leads to increased bank lending as interest rates fall, which further bring more investment. In addition, from a project selection perspective, Huang and Xu [15] argue that an economy with multi-bank system better promotes innovation when the research and development (R&D) projects include more advanced technology. However, it reduces innovation when the R&D projects include less technology. They argue that an economy with single bank system has soft budget constraints, while, an economy with multiple bank system has hard budget constraints. Consequently, corporate innovation can be fostered under both competitive and monopolistic banking structures, indicative of a nonlinear relationship between the two.

Building on early theoretical models, researchers have undertaken empirical investigations into the connection between banking market structure and corporate innovation. Carlin and Mayer [7] investigate OECD countries and find that bank competition reduces research and development (R&D) investment in developed countries, while the opposite effect is observed in developing countries. Amore et al., Chava et al., Cornaggia et al. and Deng et al. [9, 10, 16, 34] analyze how US intrastate and inter-state bank branch deregulation affect firm innovation and obtain inconsistent results. Several studies have explored the relationship between bank market structure and firm innovation in China. For instance, Zhang et al. [21] examine the non-linear relationship between bank market structure and firm innovation, highlighting that the intrinsic motivation behind this U-shaped relationship related to the monopolistic character of the banking sector. Liu and Li [18] indicate that bank competition has a positive effect on firm innovation. Xia and Liu [20] demonstrate an inverted-U relationship between bank competition and R&D investment. Huang et al. [35] point out that bank competition promotes the regional innovation in China. Xin et al. [19] find that bank deregulation on city commercial banks in China has significantly positive effect on firm innovation. Additionally, Xia and Liu [36] explore the impact of bank competition on firm green innovation in China. Tan et al. [37] illustrate that a combination of industry and finance serves as a moderating factor in the relationship between bank competition and enterprise innovation. Furthermore, Li and Peng [38] present evidence that bank price competition can effectively enhance innovation investment and output for firms.

Despite the extensive body of literature that has examined the relationship between bank market structure and firm innovation in China, there has been limited discussion about the non-linear nature of this relationship, particularly regarding its intrinsic determinants and the associated effects on firm and regional heterogeneity.

## 3. Chinese banking market structure

**Fig 1:**
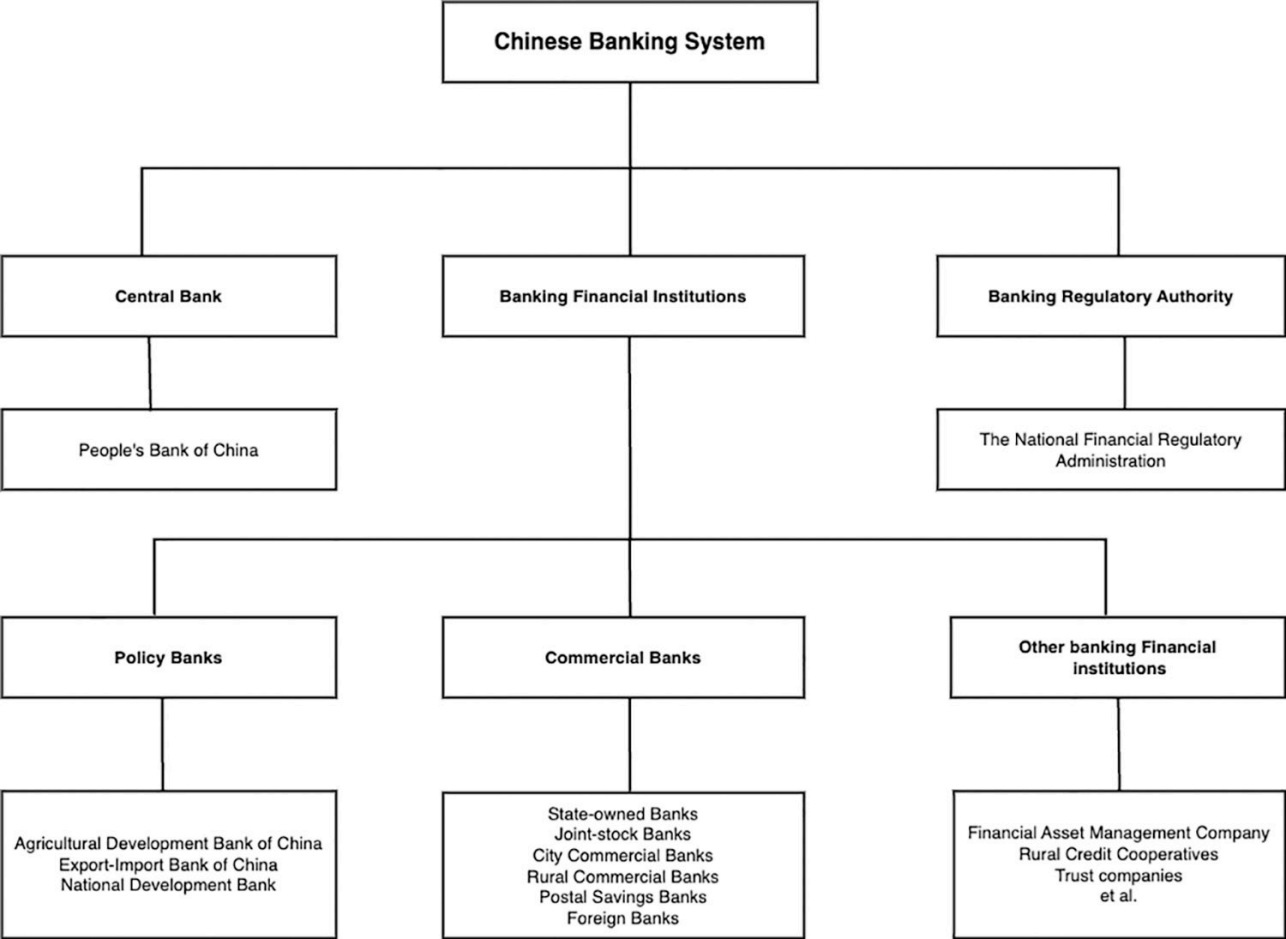
The Chinese banking system. This figure shows the structure of the Chinese banking system.

The Chinese banking system originated as a mono-bank system, with the People’s Bank of China (PBC) as the only bank. Over recent years, the Chinese banking system has undergone significant development. However, over the past three decades, the Chinese banking system has witnessed substantial transformations. Through a series of reforms, China has evolved into a banking system primarily composed of the central bank, a banking regulatory authority, policy banks, commercial banks, and various other financial institutions. Fig 1 illustrates the structure of the Chinese banking market.

The People’s Bank of China, operating as the central bank, falls under the jurisdiction of the State Council and holds responsibilities for formulating and executing monetary policies, actively managing and mitigating financial risks, maintaining financial stability, and providing financial services. The National Financial Regulatory Administration (NFRA) serves as the banking regulatory authority, overseeing and regulating banking and financial institutions. Policy banks, initiated and funded by the government, are established to support specific economic policies and objectives through financing and credit activities. Commercial banks, on the other hand, typically refer to profit-oriented banks engaged in deposit-taking, loan provision, and various intermediary services. In addition to these components, China’s banking system encompasses credit cooperatives, financial asset management companies, and other financial institutions.

Specifically, Chinese commercial banks mainly consist of state-owned banks, joint-stock banks, city commercial banks, rural commercial banks, and the Postal Savings Bank of China. As argued by Chang et al. [39], from 2002 to 2009, the Chinese banking industry gained 29.84% in total factor productivity growth.

According to data documented by the NFRA, there are five big state-owned banks in China, which include the Bank of China (BOC), the China Construction Bank (CCB), the Agricultural Bank of China (ABC), and the Industrial and Commercial Bank of China (ICBC) and the Bank of Communications (BCM). The customers of the big five state-owned firms are mainly state-owned enterprises (SOEs) and large firms. ICBC is the largest commercial bank in China. It used to be the major credit supplier for China’s manufacturing sector. BOC specializes in foreign-exchange transactions and trade finance. CCB is the major credit supplier for infrastructure projects and urban housing development, and ABC specializes in China’s agricultural sector and offers wholesale and retail banking services to farmers, township and village enterprises (TVEs), and other rural institutions. BCM is a commercial bank with multiple functions, mainly serving the development of cities. Under the pressure of international and domestic competition, the big five state-owned banks were forced to boost their financial conditions, especially asset quality, as well as improve their risk management and corporate governance. During 2005 to 2010, the five state-owned banks were partially privatized, took on minority foreign ownership, and were successfully listed in stock markets. All these reforms increased their performance [40]. The big state-owned banks have already used commercial judgment in allocating loans to private firms, especially for large firms and manufacturing firms [41].

In addition to the “big five” state-owned commercial banks, joint-stock commercial banks also operate nationwide. Due to better organizational structure, more market oriented and more efficient operation, joint-stock banks are powerful competitors with the big state-owned banks. As argued by Chang et al. [39], the joint-stock banks are the most efficient banks in China.

The third significant group in the Chinese banking market is the city commercial banks. Many of them were founded on the basis of urban credit cooperatives. The first was the Shenzhen City Commercial Bank, established in 1995. The city commercial banks’ market orientation is towards supporting the regional economy, it also focuses on financing local infrastructure and other government projects. Since 2006, city commercial banks have been allowed to operate nationally, and several of them have quickly expanded. As argued by Ferri and Chang et al. [39, 42], the city commercial banks outperform state-owned commercial banks.

Chinese rural commercial banks have mainly been founded on the basis of rural credit cooperatives. They intend to help the rural economy and provide services to agriculture-related projects. Their development is slower than that of the big state-owned banks, the joint-stock banks, and city commercial banks. The first rural commercial bank, the Zhangjiagang Rural Commercial Bank, was established in 2001.

The foreign banks developed very fast in China after China joined WTO. The proportion of foreign banks is not big. Until the end of 2006, the assets of foreign banks constitute of 2.11% of total assets of financial institutions in China.

The Postal Savings Bank of China (PSBC) is a commercial retail bank. It was established in 2009 from the State Post Bureau in 2007. It started providing loans to small and medium enterprises (SMEs) in January, 2009.

## 4. Sample, measures, and summary statistics

### 4.1 Sample

Due to the data availability, the sample period of our analysis is from 1998 to 2013. Since the dependent variables are forward for one period, the dependent variables are from 1999 to 2013 and the independent variables are from 1998 to 2012. We mainly rely on three types of data sets: the annual surveys of industrial firms conducted by the NBSC, a patent data set obtained from China’s State Intellectual Property Office (SIPO) and the data released by NFRA. The database is composed of a considerable amount of information on firms, including the characteristics, financial information and production information of firms. We use the method of Brandt et al. [43] to construct the data in a panel. The patent database includes applications information of patents and the name and address of applicants. We merge these two databases using the name and city of applicants. NFRA provides various types of information related to each branch, sub-branch of a bank and each bank. The data includes the name, type, address and the date of establishment of each sub-branch, branch and bank. From NFRA, we extract the city information of each sub-branch, branch of a bank and the type of the branch and the bank. Afterwards, we construct the bank market structure measure. The city-level control variables are obtained from the China City Statistical Yearbook. After excluding the missing values, there are 288 cities with 2,412,316 observations left. In order to eliminate the effect of the abnormal values, we winsorize the firm-level control variables with abnormal values at the 1% and 99% quantiles.

### 4.2 Innovation measure

We use the logarithm of one plus the number of patents applied for by a firm (*lnpat*) as the proxy for a firm’s innovation. The larger the number of patents, the more innovative the firms are. Following Tan et al. [44], we only include both the invention and utility model patents. In China, the invention and utility model patents are defined by SIPO as “new technical solutions proposed for a product, a process or the improvement thereof” and “new technical solutions proposed for the shape and structure of a product, or the combination thereof, which are fit for practical use,” respectively. Thus, in our basic regression, we use the logarithm of one plus the number of invention and utility model patents applied for by a firm (*lnpat*), the logarithm of one plus the number of invention model patents applied for by a firm (*lninvention*) and the logarithm of one plus the number of utility model patents applied for by a firm (*lnutility*) as dependent variables.

### 4.3 The market structure of commercial banks in China

We use the Herfindahl–Hirschman Index (HHI) to measure the market structure of Chinese commercial banks. HHI is a measure widely used to proxy the market structure of an industry. The lower the HHI, the more competitive an industry is. Following Chong et al. [45], we define the HHI in the banking industry as follows:

$${hhi\_bank}_{j,t}=\sum_{m=1}^{Mj,t}{(\#{branch}_{m}/\sum_{m=1}^{Mj,t}\#{branch}_{m})}^{2}$$ (1)

Where *Mj*,*t* is the number of commercial banks in city *j* in year *t*, and *#branch*_*m*_ represents the number of branches and sub-branches in the mth bank. The banks we cover are the big state-owned banks, joint-stock banks, city commercial banks and foreign banks. However, we do not include RCB since they mainly provide loans for agriculture in our sample period. We also exclude the PSBC because it started to providing loans to enterprises in January, 2009.

### 4.4 Control variables

Following the literature [36, 44], we control for firms’ size (*Size*), age (*lage*), return on assets (*ROA*), firms’ leverage (*leverage*) and whether a firm is an export firm (*export*). To mitigate the omitted variable bias, we also control the GDP per capita (*lgdppc*), bank density (*Bankdens*), the government expenditure on science and technology (*lnSTexp*), and the employee wages (*lnWage*) in a city. The definitions of the variables are presented in Table 1.

**Table 1:** Variable definitions.

Variables	Definition
Measure of Innovation	
*lnpat*_*i*,*t+1*_	The natural log of one plus the number of invention and utility model patents applied for by firm *i* in year *t+1*.
*lninvention*_*i*,*t+1*_	The natural log of one plus the number of invention model patents applied for by firm *i* in year *t+1*.
*lnutility*_*i*,*t+1*_	The natural log of one plus the number of utility model patents applied for by firm *i* in year *t+1*.
Measure of bank HHI and other variables in the baseline regression	
*hhi_bank*_*j*,*t*_	The Herfindahl-Hirschman Index of banking industry in the city *j* where a firm is located in year *t*.
*lgdppc*_*j*,*t*_	The natural log of one plus the GDP per capita in the city *j* where a firm is located in year *t*.
*Bankdens*_*j*,*t*_	The logarithm of all the branches and sub-branches of commercial banks excluding the rural commercial banks and post-savings banks in the city *j* where a firm is located in year *t*.
*lnSTexp*_*j*,*t*_	The logarithm of government expenditure on science and technology in the city *j* where a firm is located in year *t*.
*lnWage*_*j*,*t*_	The logarithm of employee wages in the city *j* where a firm is located in year *t*.
*export*_*i*,*t*_	A dummy variable that equals one if the total export for a firm *i* is greater than zero in year *t*, and zero otherwise.
*size*_*i*,*t*_	The natural log of the total assets of firm *i* in year *t*.
*leverage*_*i*,*t*_	A firm *i*’s leverage that is defined as total liabilities divided by total assets in year *t*.
*lage*_*i*,*t*_	The natural log of one plus the difference between year t and the founding year of a firm *i*.
*ROA*_*i*,*t*_	A firm *i*’s return on asset that is defined as profit divided by total asset of the firm *i* in year *t*.

### 4.5 Summary statistics

In Table 2, we summarize the variables in the sample period used in our baseline regression. More than half of the observations have zero patents. The mean number of patents that a sample firm applied for in one year is 0.1007. The maximum number of patents that a firm applied for in one year is 6110. The mean and median of *hhi_bank* for a city is 0.1929 and 0.1831, respectively. The control variables are also summarized in the table. From 1998 to 2012, a firm has average assets of 22,388,439 RMB [exp(10.0163)*1000], an average ROA of 0.0994, and an average age of 7.5610 years[exp(2.023)-1]. The mean of *leverage* is 0.5568 in a year. Moreover, on average, 31.39% of the observations have exports. Furthermore, the average GDP per capita of a city is 31,758 RMB [exp (10.3659)]. On average, there are 375 [exp (5.9274)] branches and sub-branches of commercial banks (excluding rural commercial banks and post savings banks) per city. The government expenditure on science and technology averages at 119.1675 million RMB [exp (9.3857) * 10000], and the average employee wages in the city are 23,237 RMB [exp (10.0535)].

**Table 2:** Summary statistics.

	(1)	(2)	(3)	(4)	(5)
Variables	mean	sd	min	p50	Max
*lnpat*_*i*,*t+1*_	0.1007	0.4439	0.0000	0.0000	8.7178
*lninvention*_*i*,*t+1*_	0.0473	0.2833	0.0000	0.0000	8.6810
*lnutility*_*i*,*t+1*_	0.0756	0.3693	0.0000	0.0000	7.2196
*hhi_bank*	0.1929	0.0567	0.0779	0.1831	0.6698
*lgdppc*	10.3659	0.9504	4.3619	10.3547	13.0176
*Bankdens*	5.9247	0.9444	2.4849	5.8916	7.9025
*lnSTexp*	9.3857	2.3440	0.0000	9.3701	14.7133
*lnWage*	10.0535	0.5617	2.2834	10.1150	11.8284
*export*	0.3139	0.4641	0.0000	0.0000	1.0000
*size*	10.0163	1.2881	7.9997	9.8745	12.6639
*leverage*	0.5568	0.2597	0.0761	0.5749	0.9773
*lage*	2.0230	0.7062	0.6931	2.0794	3.3673
*ROA*	0.0994	0.1502	-0.0511	0.0435	0.5867

**Fig 2:**
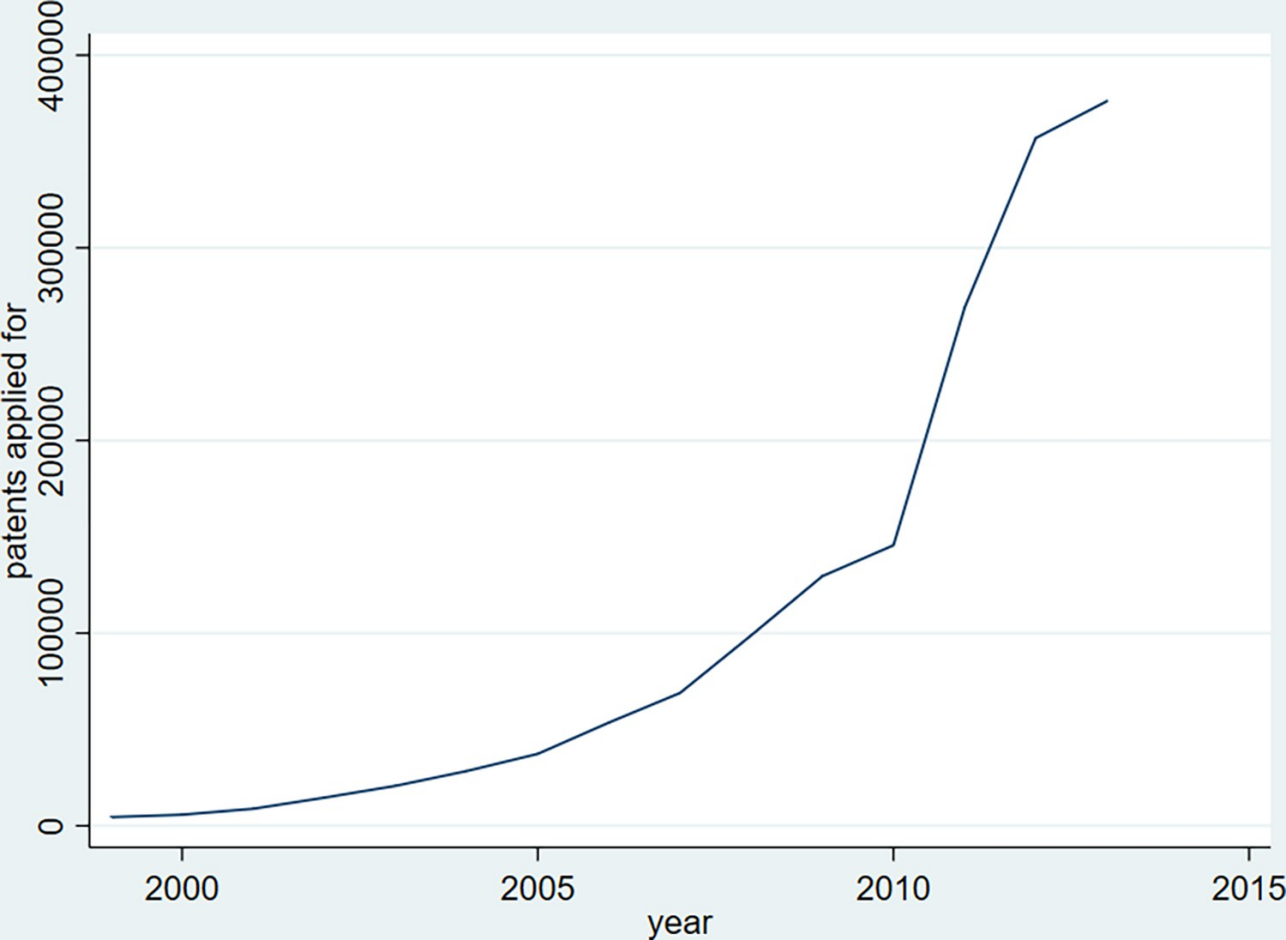
The number of patents applied for. This figure shows the total number of patents applied for each year from 1999 to 2013. The firms in our sample are from 288 prefecture-level cities in China.

Fig 2 plots the annual number of patents applied for by all firms from 1999 to 2013. The figure shows a steady increase in the number of patents applied for.

## 5. Results

### 5.1 The empirical model

The model we estimate is the following:

$$Y_{i,t+1}=\beta_{1}+\beta_{2}\times {hhi\_bank}_{j,t}+\beta_{3}\times {{hhi\_bank}^{2}}_{j,t}+\lambda^{\prime }\times X_{i,t}+\gamma^{\prime }\times D_{j,t}+u_{i}+\alpha_{t}+\varepsilon_{i,t},\;i=1,\ldots ,N;t=1,\ldots ,T$$ (2)

where *Y*_*i*,*t+1*_ is the logarithm of 1 plus the number of patents (invention and/or utility model) applied for firms *i* in year *t+1*; *hhi_bank*_*j*,*t*_ is the bank market structure in city *j* at year *t*; *hhi_bank*^*2*^_*j*,*t*_ is the square of *hhi_bank*_*j*,*t*_. *X*_*i*,*t*_ is a matrix of firm-level control variables for firms *i* at year *t*; *D*_*j*,*t*_ is a matrix of city-level control variables for city *j* at year *t*; *μ*_*i*_ and *α*_*t*_ are firm and year fixed effect, respectively, *ε*_*i*,*t*_ is the error term; *β*_*2*_ and *β*_*3*_ are coefficients for *hhi_bank*_*j*,*t*_ and *hhi_bank*^*2*^_*j*,*t*_, respectively. *λ* and γ are coefficient vector for firm-level control variables and city-level control variables, respectively. *β*_*1*_ is the constant term. *β*_*2*_ and *β*_*3*_ denote the effect of the bank market structure on firms’ innovation outputs in a city. We include the firm and city controls to reduce the omitted variable problem and the firm fixed effect to partially solve the spatial sorting endogeneity problem.

### 5.2 Baseline results

In this subsection, we provide results for the effect of bank market structure on firms’ innovation outputs.

Table 3 shows the results of the basic model. In columns (1) and (2), we present the effect of bank market structure on the number of patents (*lnpat)* without and with the control variables, respectively. To check the effect of bank market structure on different types of patents, we conduct the regression of bank market structure on number of invention patents (*lninvention*), and number of utility model patents (*lnutility*). The results are shown in columns (3) and (4). Our results indicate that the coefficients of *hhi_bank* in all columns are significantly negative and the coefficients of *hhi_bank*^*2*^ in all columns are significantly positive, meaning that there is a U-shaped relationship between bank market structure and the number of patents applied for by a firm. In particular, the coefficients of *hhi_bank* and *hhi_bank*^*2*^ in column (2) is -0.8517 and 1.2998, respectively. Therefore, based on these coefficients, the peak of *hhi_bank* can be calculated as 0.3276. According to our findings, in a highly concentrated banking markets, increased bank competition leads to a decrease in the number of patents, including invention and utility model patents. In contrast, when bank market structure is more competitive, increased bank competition is associated with an increase in the number of patents, including invention and utility model patents.

**Table 3:** The effect of bank market structure on firm innovation.

	(1)	(2)	(3)	(4)
	*lnpat*_*i*,*t+1*_	*lnpat*_*i*,*t+1*_	*lninvention*_*i*,*t+1*_	*lnutility*_*i*,*t+1*_
*hhi_bank*	-1.0542***	-0.8517***	-0.6853***	-0.5589***
	(0.0658)	(0.0665)	(0.0443)	(0.0567)
*hhi_bank* ^2^	1.5176***	1.2998***	0.9279***	0.9036***
	(0.1012)	(0.1003)	(0.0695)	(0.0862)
*lgdppc*		0.0033	0.0064***	0.0000
		(0.0023)	(0.0015)	(0.0019)
*Bankdens*		0.0107***	-0.0075***	0.0138***
		(0.0031)	(0.0020)	(0.0027)
*lnSTexp*		0.0079***	0.0057***	0.0054***
		(0.0004)	(0.0003)	(0.0003)
*lnWage*		-0.0061*	0.0127***	-0.0149***
		(0.0036)	(0.0025)	(0.0030)
*export*		0.0461***	0.0238***	0.0364***
		(0.0015)	(0.0010)	(0.0012)
*Size*		0.0356***	0.0147***	0.0269***
		(0.0009)	(0.0006)	(0.0007)
*leverage*		-0.0092***	-0.0102***	-0.0029*
		(0.0018)	(0.0012)	(0.0016)
*lage*		-0.0753***	-0.0493***	-0.0557***
		(0.0027)	(0.0019)	(0.0023)
*ROA*		-0.0034	-0.0055***	-0.0055**
		(0.0027)	(0.0018)	(0.0022)
Constant	0.2427***	-0.1108**	-0.1107***	-0.0026
	(0.0090)	(0.0514)	(0.0352)	(0.0431)
Firm Fe	Yes	Yes	Yes	Yes
Year Fe	Yes	Yes	Yes	Yes
Observations	2412316	2412316	2412316	2412316
Adjusted R-squared	0.4557	0.4582	0.4314	0.4179

Note: This table shows the estimation of our basic model. The dependent variable in columns (1) and (2) is the natural log of one plus the number of invention and utility model patents applied for by a firm in year *t+1*. The dependent variable in column (3) is the natural log of one plus the number of invention patents applied for by a firm in year *t+1*. The dependent variable in column (4) is the natural log of one plus the number of utility model patents applied for by a firm in year *t+1*. The *hhi_bank* is the Herfindahl-Hirschman Index of banking industry in the city *j* where a firm is located in year *t*. The *hhi_bank*^*2*^ is the square of *hhi_bank*. The definitions of the control variables are presented in Table 1. We estimate the coefficients using fixed effect regression with year and firm fixed effects clustered at the firm level in columns (1) to (4). Standard errors are presented in parentheses.

*, **, and *** denote significance at the 10%, 5%, and 1% levels, respectively.

In contrast to Xia and Liu [20], who have identified an inverted-U relationship between bank competition and corporate R&D investments, our study reveals a U-shaped relationship between bank market structure and firm innovation output. Unlike Liu and Li [18], who mainly concentrate on the linear relationship between bank competition and firm innovation output, our research centers on exploring the non-linear relation between bank market structure and firm innovation output.

### 5.3 Robustness check

To ensure the reliability and validity of our findings, we undertake several robustness checks. Firstly, we conduct a U-shaped test to confirm the existence of a non-linear relationship between bank market structure and firm innovation. Secondly, we employ an instrumental variable (IV) regression to verify the credibility of our results. Thirdly, we check whether our results remain by testing alternative measures for bank market structure. Fourthly, we investigate the robustness of our findings by employing alternative proxies for innovation. Finally, to confirm the generality of our results, we adopt the A-share listed sample to extend the sample period until 2019 excluding the COVID-19 pandemic period.

#### 5.3.1 U-shape test

To verify the veracity of the U-shaped relationship between bank market structure and firm innovation, we employ the U-shape test proposed by Lind and Mehlum [17]. If the U-shaped relationship holds, the U curve should exhibit a negative slope at the lower bound and a positive slope at the upper bound, with only one inflection point. The null and alternative hypotheses for the U test, based on our basic model, are as follows:

$$H0:\;\beta_{2}+{2\beta }_{3}\times {min\;(hhi\_bank}_{j,t})>=0\;and\;\beta_{2}+2\beta_{3}\times {max\;(hhi\_bank}_{j,t})<=0$$ (3)

vs.


$$H1:\;\beta_{2}+{2\beta }_{3}\times {min\;(hhi\_bank}_{j,t})<0\;and\;\beta_{2}+2\beta_{3}\times {max\;(hhi\_bank}_{j,t})>0$$

The rejection area is as follows:

$$\{(\beta_{2},\beta_{3}):\frac{\beta_{2}+2\beta_{3}{min\;(hhi\_bank}_{j,t})}{\sqrt{s_{22}+4\min(hhi\_bank_{j,t})s_{23}+4(\min(hhi\_bank_{j,t}{))}^{2}s_{33}}}<-t_{\partial }$$

$$and\;\frac{\beta_{2}+2\beta_{3}{max\;(hhi\_bank}_{j,t})}{\sqrt{s_{22}+4\max(hhi\_bank_{j,t})s_{23}+4(\max(hhi\_bank_{j,t}{))}^{2}s_{33}}}>t_{\partial }\}$$ (4)

where *s*_*22*_, *s*_*23*_, *s*_*33*_ are the estimated variances and covariance of *β*_*2*_ and *β*_*3*_, respectively. The outcome of the U-test is displayed in Table 4. The results of the U-test indicate that the overall test of presence of a U shape rejects the null hypothesis, which means that there is indeed a U-shaped relationship between *hhi_bank* and *lnpat*.

**Table 4:** The U-shape test.

Dependent variable: *lninvention*_*i*,*t+1*_	Without controls	With controls
Slope of *hhi_bank*	-1.0542	-0.8517
Slope of *hhi_bank*^2^	1.5176	1.2998
Slope at lower bound	-0.8178	-0.6492
Slope at upper bound	0.9786	0.8893
Overall test of presence of a U shape(t-value)	12.65	11.61
Overall test of presence of a U shape(p-value)	0.0000	0.0000

Note: This table shows the U-shape test. The dependent variable is the natural log of one plus the number of invention and utility model patents applied for by a firm in year *t+1*. The *hhi_bank* is the Herfindahl-Hirschman Index of banking industry in the city *j* where a firm is located in year *t*. The *hhi_bank*^2^ is the square of *hhi_bank*. *, **, and *** denote significance at the 10%, 5%, and 1% levels, respectively.

#### 5.3.2 Instrumental variable regression

Given that the bank market structure variable is measured at the city level and the dependent variable is at the firm level, and that we lag our independent variables by one period, reverse causality is not a significant issue in our regression analysis. However, omitted variable bias may still arise. To address this concern and enhance the reliability of our results, we employ an IV method. Following Chong et al. [45], we employ the average of the variable *hhi_bank* in cities except the local city within the same province as our instrument. For municipalities directly under the central government, we select other municipalities under the central government or adjacent sub-provincial cities as the instrument. Specifically, we use the average of *hhi_bank*_*i*,*t*_ of Nanjing, Hangzhou and Ningbo as IV for that of Shanghai, *hhi_bank*_*i*,*t*_ of Tianjin as IV for that of Beijing, *hhi_bank*_*i*,*t*_ of Beijing as IV for that of Tianjin, the average of *hhi_bank*_*i*,*t*_ of Chengdu, Wuhan and Xi’an for that of Chongqing.

Our instrumental variable satisfies the conditions of exclusion restriction and relevance. First, given the city-specific nature of the Chinese credit market [46], bank branches typically do not conduct business with firms in other cities. Since our instrumental variable solely encompasses the bank market structures of cities except the local area, it reduces the likelihood of the instrumental variable being correlated with the error term affecting the innovation outputs of local firms (exclusion restriction). Second, the bank market structure in cities within the same province may be correlated due to similar geographic and social characteristics (relevance). Furthermore, the first-stage F-test demonstrates statistical significance at the 1% level, indicating that the instrument is significantly correlated with the bank market structure variable in local cities.

The columns (1)-(3) of Table 5 present the results of the IV regression. Following Jin et al. [47], we perform a second-stage estimation by replacing *hhi_bank* and *hhi_bank*^*2*^ in our basic model with their predicted values obtained from the first-stage IV regression. The results in columns (1)-(3) indicate that the coefficients of the instrumented *hhi_bank* and *hhi_bank*^*2*^ are significantly negative and positive, respectively. These findings suggest a causal and U-shaped relationship between bank market structure and firm innovation. Thus, the IV analysis reinforces our belief that there is a U-shaped relationship between bank market structure and firm innovation and the relation is causal.

**Table 5:** The effect of bank market structure on firm innovation-IV regression.

	(1)	(2)	(3)
	*lnpat*_*i*,*t+1*_	*lninvention*_*i*,*t+1*_	*lnutility*_*i*,*t+1*_
*hhi_bank*	-8.3727***	-5.3931***	-5.8623***
	(0.4008)	(0.2824)	(0.3284)
*hhi_bank* ^2^	4.8931***	2.9756***	3.5178***
	(0.3291)	(0.2325)	(0.2710)
Constant	4.2291***	2.6713***	3.0322***
	(0.2742)	(0.1901)	(0.2253)
Controls	Yes	Yes	Yes
Firm Fe	Yes	Yes	Yes
Year Fe	Yes	Yes	Yes
Observations	2412316	2412316	2412316
Adjusted R-squared	0.4587	0.4318	0.4182
The p-value of first stage F test	0.0000		

Note: The columns (1) to (3) in this table show the estimation of the IV regression. The dependent variable in columns (1) to (3) is the natural log of one plus the number of invention and utility model patents applied for by a firm in year *t+1*. The *hhi_bank* is the Herfindahl-Hirschman Index of banking industry in the city *j* where a firm is located in year *t*. The *hhi_bank*^*2*^ is the square of *hhi_bank*. The instrumental variable is *hhi_bank_iv*. The *hhi_bank_iv* is the average of the variable *hhi_bank* in cities except the local city in the same province. For the municipalities directly under the central government, we use other municipalities under the central government or sub-provincial cities adjacent as the instrument. The city of Xining in Qinghai Province is excluded from the regression in this Table due to the lack of sufficient observations. The definitions of the control variables are presented in Table 1. We estimate the coefficients using fixed effect regression with year and firm fixed effects clustered at the firm level. Standard errors are presented in parentheses.

*, **, and *** denote significance at the 10%, 5%, and 1% levels, respectively.

#### 5.3.3 Other proxies of bank market structure

As the number of foreign banks is relatively small during 1998 to 2013 in China, we further check our results using the Herfindahl-Hirschman Index of banking industry that only includes the big state-owned banks, joint-stock banks and city commercial banks (*hhi2_bank*) as the proxy of bank market structure.

The result is presented in column (1) of Table 6. In columns (1), the market structure of Chinese commercial banks is measured by the Herfindahl-Hirschman Index of banking industry that only includes the big state-owned banks, joint-stock banks and city commercial banks. The result show that the coefficients of *hhi_bank* and *hhi_bank*^*2*^ are still significantly negative and positive, respectively, which is consistent with our main results.

**Table 6:** The robustness check of bank market structure on firm innovation.

	(1)	(2)	(3)
	*lnpat*_*i*,*t+1*_	*lnpat*_*i*,*t+2*_	*lnpat*_*i*,*t+1*_
*hhi2_bank*	-0.7278***		
	(0.0658)		
*hhi*2*_bank*^2^	1.1462***		
	(0.0991)		
*hhi_bank*		-0.9232***	-6.7422**
		(0.0770)	(3.4034)
*hhi_bank* ^2^		1.3844***	12.0085*
		(0.1152)	(7.1412)
Constant	-0.1397***	-0.1564***	-11.6035***
	(0.0508)	(0.0588)	(2.1014)
Controls	Yes	Yes	Yes
Firm Fe	Yes	Yes	Yes
Year Fe	Yes	Yes	Yes
Observations	2412316	2054406	18817
Adjusted R-squared	0.4582	0.4851	0.7382

Note: The sample of column (4) only include A-share listed companies in China from 2003–2019. The dependent variable in columns (1) and (3) is the natural log of one plus the number of invention and utility model patents applied for by a firm in year *t+1*. The dependent variable in columns (2) is the natural log of one plus the number of invention and utility model patents applied for by a firm in year *t+2*. The *hhi_bank* is the Herfindahl-Hirschman Index of banking industry in the city *j* where a firm is located in year *t*. The *hhi_bank*^*2*^ is the square of *hhi_bank*. The *hhi2_bank* is the Herfindahl-Hirschman Index of banking industry that includes the big state-owned banks, joint-stock banks and city commercial banks in the city *j* where a firm is located in year *t*. The *hhi2_bank*^*2*^ is the square of *hhi2_bank*. The control variables in columns (1) and (2) include *lgdppc*, *Bankdens*, *lnSTexp*, *lnWage*, *export*, *size*, *leverage*, *lage* and *ROA*. The definitions of the control variables in columns (1) and (2) are presented in Table 1. The control variables in columns (3) are *lgdppc*, *Bankdens*, *lnSTexp*, *lnWage*, *export*, *size*, *leverage*, *lage*, *ROA* and *subsidy*. The *subsidy* is defined the ratio of the government’s subsidy to total assets. The definitions of the other control variables in column (3) are presented in Table 1. We estimate the coefficients using fixed effect regression with year and firm fixed effects clustered at the firm level. Standard errors are presented in parentheses.

*, **, and *** denote significance at the 10%, 5%, and 1% levels, respectively.

#### 5.3.4 Other proxy for innovation outputs

In this part, we utilize the number of patents applied for firms *i* in year *t+2* as a proxy for innovation. The result is reported in column (2) of Table 6 and it is consistent with our previous findings.

#### 5.3.5 Alternative sample period

Owing to data availability, our baseline findings are based on the industrial database spanning from 1998 to 2013. To mitigate concerns regarding the sample period and validate the generalizability of our results, we adopt the A-share listed sample to extend the sample period until 2019 excluding the influence of the COVID-19 pandemic. Owing to data availability constraints within the CSMAR database, the initial year for our sample of A-share listed companies commences in 2003. The results are displayed in column (3) of Table 6, illustrating a U-shaped relationship between bank market structure and innovation. This discovery provides further confirmation of the reliability of our baseline results.

## 6. Channel

The purpose of this section is to investigate whether the financing channel is driving the U-shaped relationship between bank market structure and firm innovation. Existing literature presents two theoretical predictions regarding whether and how bank market structure supports firm innovation through financing channel. A competitive market structure may stimulate firm innovation by facilitating access to finance through efficient banking systems [11]. In contrast, a more centralized banking market could boost innovation output as banks, aiming to mitigate informational asymmetries, are more likely to cultivate long-term relationships, thereby providing consistent financial support for innovative projects [13]. Therefore, we hypothesize that the financing channel may play a crucial role in driving our primary findings. We investigate the financing channel from two perspectives, including external financial dependence and fixed asset ratios.

### 6.1 External finance dependence

Since innovation activities require financing, firm innovation can be promoted by alleviating firms’ financial constraint. It is widely accepted that if financing is one of the channels through which financial development affects firm innovation, firms operating in industries with high external financial dependency are likely to be more affected than those in industries with low external financial dependency. This is also applicable in the case of the bank market structure, meaning that if the bank market structure impacts firm innovation through financing channels, the marginal benefit of funding for a high EFD firm is expected to be greater than that for a low EFD firm. Consequently, the innovation output of high EFD firms should be more influenced by bank market structure than that of low EFD firms.

To investigate this hypothesis, we add the interaction of bank market structure and EFD into the basic model. Since EFD could be endogenous to a country’s financial development when constructed with local data, several studies [2, 48–50] have adopted US firm data to construct EFD, as the US has one of the most mature financial markets in the world and firms’ optimal external finance dependence can be reflected. In our paper, we follow the approach. Specifically, we calculate the EFD of a firm using US firm data as follows:

$${EFD}_{i,t}=\frac{{capital\;expenditure}_{i,t}-{funds\;from\;operations}_{i,t}}{{capital\;expenditure}_{i,t}}$$ (5)

When the data for *funds from operations* are missing, we calculate them as follows:

*Funds from operations*_*i*,*t*_ *= Income Before extraordinary Item*_*i*,*t*_ *+ Depreciation and Amortization*_*i*,*t*_ *+ Deferred Taxes*_*i*,*t*_ *+ (Equity in Net Loss/Earnings)*_*i*,*t*_ *+(Sale of Property*, *Plant and Equipment and Investments gain/loss)*_*i*,*t*_ *+ Funds from operations other*_*i*,*t*_. The data used in this study covers the period from 1990 to 1997 and was obtained from Compustat. We took the EFD of a firm as the median of the firm’s yearly external finance dependence. The EFD of an industry in the US was calculated as the median EFD of the firms within that industry. To determine the external finance dependence of the Chinese industry, we matched the 2-digit 1997 US Standard Industry Classification (SIC) code with the 2-digit 2002 China Industry Classification (CIC) code.

The result is presented in Table 7. The coefficient of interaction of bank market structure and EFD (hhi_bank*EFD) in column (1) is significantly negative and the coefficient of *hhi_bank*^2^*EFD in column (1) is significantly positive. The findings indicate that firms with high EFD are more sensitive to bank market structure than those with low EFD. Specifically, in a highly concentrated banking market, companies in high EFD industries experience a greater decrease in innovation as bank competition increases. This may be due to reduced access to credit, which hampers their ability to innovate. In contrast, in a more competitive stage, the innovation of firms in high EFD industries increases more as bank competition increases. Overall, the results support the idea that financing channel is indeed driving the U-shaped relationship between bank market structure and firm innovation.

**Table 7:** The effect of bank market structure on firm innovation- financing channel.

	(1)	(2)
	*lnpat*_*i*,*t+1*_	*lnpat*_*i*,*t+1*_
*hhi_bank*	-2.8068***	-1.2167***
	(0.1172)	(0.0959)
*hhi_bank* ^2^	3.6842***	1.7205***
	(0.1939)	(0.1777)
*hhi_bank* * *EFD*	-3.8196***	
	(0.1540)	
*hhi_bank* ^2^ * *EFD*	4.6315***	
	(0.2451)	
*EFD*	0.5298***	
	(0.0211)	
hhi_bank* *fixratio*		1.1308***
		(0.1404)
*hhi_bank*^2^* *fixratio*		-1.3740***
		(0.2779)
*fixratio*		-0.1667***
		(0.0174)
Constant	0.1602***	-0.0780
	(0.0525)	(0.0520)
Controls	Yes	Yes
Firm Fe	Yes	Yes
Year Fe	Yes	Yes
Observations	2412316	2412075
Adjusted R-squared	0.4591	0.4583

Note: The dependent variable in all columns is the natural log of one plus the number of invention and utility model patents applied for by a firm in year *t+1*. The *hhi_bank* is the Herfindahl-Hirschman Index of banking industry in the city *j* where a firm is located in year *t*. *hhi_bank*^*2*^ is the square of *hhi_bank*. The *EFD* is the external financial dependence of an industry that a firm belongs to. The *fixratio* is defined as fixed assets divided by total assets. The definitions of the control variables are presented in Table 1. We estimate the coefficients using fixed effect regression with year and firm fixed effects clustered at the firm level. Standard errors are presented in parentheses.

*, **, and *** denote significance at the 10%, 5%, and 1% levels, respectively.

### 6.2 Fixed asset ratio

Banks often prefer firms with higher fixed asset ratio since fixed assets can be treated as collateral, thereby reducing lending risk. Consequently, the bank loans for firms with higher fixed asset ratios exhibit less variation than those for firms with lower fixed asset ratios. If the bank market structure affecting the firms’ innovation through financing channel, the firms with low fixed asset ratio should be more affected than those with high fixed asset ratio. To test this conjecture, we construct a variable fixratio that is defined as the ratio of fixed assets to total assets. Then we add interaction terms of *hhi_bank*, *hhi_bank*^*2*^ and *fixratio* into the regression. The result in Table 7 shows that the coefficients of both interaction terms have opposite signs to those of the *hhi_bank* and *hhi_bank*^*2*^ variables. Specifically, the coefficient of hhi_bank*fixratio is significantly positive and the coefficient of *hhi_bank*^2^*fixratio is significantly negative. These findings suggest that the bank market structure has a greater effect on firms with low fixed asset ratios than on those with high fixed asset ratios.

In summary, the findings presented in Section 6 establish that the financing channel is the fundamental mechanism underpinning the U-shaped relationship between bank market structure and firm innovation. In contrast to Zhang et al. [21], who argue that the monopolistic feature underlying the change of the bank market structure is the intrinsic driver of the U-shaped relationship between bank market structure and firm innovation, our results indicate that the U-shaped relationship between bank market structure and firm innovation is primarily propelled by the finance channel.

## 7. Further analysis

In the previous sections, we have identified a U-shaped relationship between bank market structure and firm innovation, wherein the financing channel serves as the primary driver of this relationship. In this section, we further examine the characteristics of firms and cities which drive the results.

### 7.1 Types of firms

In the introduction part, we illustrate that it is possible that when bank market structure goes from concentration to competition, the long-term relationship between banks and firms may initially suffer before more firms gain access to loans. Therefore, the average innovation of firms initially decreases and subsequently increases. We expect this effect is particularly relevant for small firms and innovative firms. Small firms have a short credit history and possess relatively more soft information, which renders them less attractive to banks. Additionally, innovative firms are generally considered riskier due to the uncertain and volatile investment payoffs associated with their innovative activities, which makes them less preferred by banks. However, when banks are able to extract more rents from small firms and innovative firms or are compelled to extend loans to less preferred firms, small firms and innovative firms can potentially acquire more financial support and hence generate more innovation output. In contrast to small firms, large firms are generally preferred by banks due to their long credit history and more hard information, so they are less susceptible to changes in bank market structure. In the meantime, since the less innovative firms have less risk compared to innovative firms, they could be less discriminated by banks. Moreover, the less innovative firms might have less incentive to engage in innovation. Therefore, the innovation output of less innovative firms might be less affected by the bank market structure than that of innovative firms from the perspective of financial support and innovation incentive. In this section, we check whether the U-shaped relationship between bank market structure and innovation exits in the samples with small firms and innovative firms, respectively.

We begin by partitioning the sample into two groups based on the size of the firms as defined by the standards provided by NBSC. The NBSC categorizes firms into four sizes: large, middle, small, and micro. Since there are no micro-firms in our sample and the middle-sized firms are relatively small, we combine middle-sized firms and small-sized firms. We compare the effects of bank market structure on small firms and large firms by presenting the results for the small-firm group and the large-firm group respectively. Furthermore, to investigate whether innovative firms are more affected by bank market structure than non-innovative firms, we divide the sample into groups with high-tech and low-tech firms. A high-tech firm is defined as a firm whose CIC code is in the list of classifications of national high-tech industries for the manufacturing industry, while a low-tech firm is defined as a firm whose CIC code of the firm is not in the list of classifications of national high-tech industries for the manufacturing industry.

**Fig 3:**
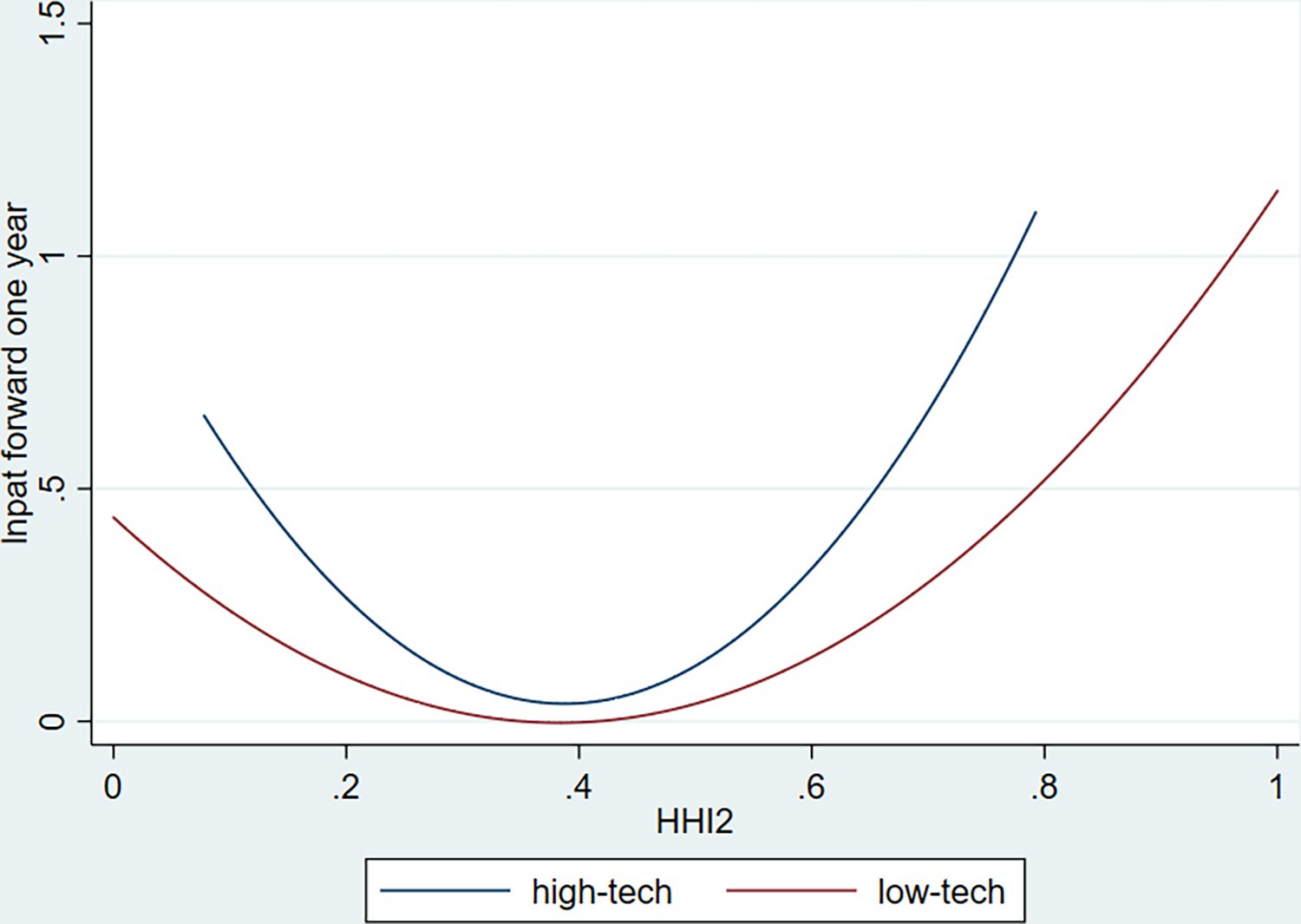
The U-shaped curve for high-tech firms and low-tech firms. This figure shows the U-shaped curve for high-tech firms and low-tech firms. The variable in Y-axis is the natural log of one plus the number of invention and utility model patents applied for by a firm in year t + 1. The variable in X-axis is the Herfindahl-Hirschman Index of banking industry in the city j where a firm is located in year t.

Table 8 presents the results for the small-firm group, large-firm group, high-tech firm group and low-tech firm group. The results in columns (1) and (2) show that the coefficients of the *hhi_bank* and *hhi_bank*^*2*^ for small firms are both significant, but the coefficients of the *hhi_bank* and *hhi_bank*^*2*^ for large firms are insignificant. The results in column (4) and (5) reveal *that the coefficients of hhi_bank* and *hhi_bank*^*2*^ for both high-tech firms and low-tech firms are significant. To compare the effect of bank market structure on the innovation for high-tech and low-tech firms, we plot the U-shaped curve for these two groups in Fig 3. Fig 3 demonstrates that the U-shaped curve for high-tech firms is steeper than that for low-tech firms, suggesting that high-tech firms are more affected by bank market structure than low-tech firms. Our findings indicate that the U-shaped relationship between bank market structure and innovation is primarily driven by small firms and innovative firms, which supports our hypothesis and further advances the understanding of the non-linear relationship of bank market structure and firm innovation.

**Table 8:** The effect of bank market structure on firm innovation- types of firms.

	(1)	(2)	(3)	(4)
	Small firms	Large firms	High-tech firms	Low-tech firms
	*lnpat*_*i*,*t+1*_	*lnpat*_*i*,*t+1*_	*lnpat*_*i*,*t+1*_	*lnpat*_*i*,*t+1*_
*hhi_bank*	-0.7628***	0.3585	-1.1005***	-0.6867***
	(0.0563)	(1.3878)	(0.3880)	(0.0657)
*hhi_bank* ^2^	1.2173***	-1.5469	1.8625***	1.0617***
	(0.0751)	(2.6530)	(0.5568)	(0.1001)
Constant	-0.2434***	0.4932	-0.6291***	-0.2378***
	(0.0473)	(0.8343)	(0.2425)	(0.0499)
Controls	Yes	Yes	Yes	Yes
Firm Fe	Yes	Yes	Yes	Yes
Year Fe	Yes	Yes	Yes	Yes
Observations	2268569	46780	175321	2232978
Adjusted R-squared	0.4051	0.6795	0.5568	0.4331

Note: This table shows the estimation of our basic model. The dependent variable in all columns is the natural log of one plus the number of invention and utility model patents applied for by a firm in year *t+1*. The *hhi_bank* is the Herfindahl-Hirschman Index of banking industry in the city *j* where a firm is located in year *t*. The *hhi_bank*^*2*^ is the square of *hhi_bank*. The *highTech* is a dummy variable that is defined as one for the firm that is in the list of classifications of national high-tech industries for the manufacturing industry in China and zero otherwise. The definitions of the control variables are presented in Table 1. We estimate the coefficients using fixed effect regression with year and firm fixed effects clustered at the firm level. Standard errors are presented in parentheses.

*, **, and *** denote significance at the 10%, 5%, and 1% levels, respectively.

### 7.2 Types of cities

#### 7.2.1 Intellectual property rights protection

A favorable IPR protection environment can protect firm innovation, thereby motivating firms to engage in innovation activities. As such, we anticipate that the impact of bank market structure on firm innovation is more pronounced in regions with better IPR protection. Following Dong et al. [51], we use provincial IPR protection index computed by Fan et al. and Wang et al. [52, 53] to measure the degree of IPR protection. We construct a variable, *highIPR*, to represent the IPR protection level for a province. Specifically, the *highIPR* is defined as one for a province with an IPR protection index above the median of all province in the current year, and zero otherwise. To verify our conjecture, we incorporate the interaction terms of *hhi_bank*, *hhi_bank*^*2*^ and *highIPR* into the baseline regression. Additionally, we control for *highIPR* itself in our regression. The result is presented in the column (1) of Table 9. The result indicates that the coefficients of the interaction term of *hhi_bank* and *highIPR* and the interaction term of *hhi_bank*^*2*^ and *highIPR* are significantly negative and positive, respectively. This suggests that the U-shaped effect of bank market structure on firm innovation is more prominent in provinces with better IPR protection.

**Table 9:** The effect of bank market structure on firm innovation- types of cities.

	(1)	(2)
	*lnpat*_*i*,*t+1*_	*lnpat*_*i*,*t+1*_
*hhi_bank*	-0.4267***	-0.6500***
	(0.0937)	(0.0798)
*hhi_bank* ^2^	0.7150***	1.0284***
	(0.1168)	(0.1212)
*hhi_bank* * *highIPR*	-0.5302***	
	(0.0925)	
*hhi_bank* ^2^ * *highIPR*	0.7882***	
	(0.1256)	
*highIPR*	0.1002***	
	(0.0150)	
hhi_bank* *highOpen*		-0.2983***
		(0.0636)
*hhi_bank*^2^* *highOpen*		0.3841***
		(0.0940)
*highOpen*		0.0538***
		(0.0095)
Constant	-0.1759***	-0.1519***
	(0.0521)	(0.0526)
Controls	Yes	Yes
Firm Fe	Yes	Yes
Year Fe	Yes	Yes
Observations	2412316	2385083
Adjusted R-squared	0.4583	0.4587

Note: The dependent variable in all columns is the natural log of one plus the number of invention and utility model patents applied for by a firm in year *t+1*. The *hhi_bank* is the Herfindahl-Hirschman Index of banking industry in the city *j* where a firm is located in year *t*. *hhi_bank*^*2*^ is the square of *hhi_bank*. The *highIPR* is defined as one if a firm is located in province where the provincial IPR protection index is above the median of overall province in year *t*, and zero otherwise in year *t*. The *highOpen* is a dummy variable that equals one if a firm is located in a city where the ratio of FDI to GDP is larger than the median of overall city in year *t*, and zero otherwise in year *t*. The definitions of the control variables are presented in Table 1. We estimate the coefficients using fixed effect regression with year and firm fixed effects clustered at the firm level. Standard errors are presented in parentheses.

*, **, and *** denote significance at the 10%, 5%, and 1% levels, respectively.

#### 7.2.2 Degree of opening-up

Foreign direct investment (FDI) plays a critical role in China’s opening-up progress [54]. The higher the degree of openness to foreign markets, the stronger the FDI technology spillover effect [55] and the greater the ability of firms to generate innovation output. Therefore, we postulate that the influence of bank market structure on firm innovation may be greater in cities with higher degree of openness to foreign markets, compared to those with lower degree of openness to foreign markets. We use the ratio of FDI to GDP to measure the degree of openness to foreign markets of a city. Then we construct a dummy variable, *highOI*, to represent the level of openness to foreign markets for a city. The variable *highOI* is defined as a binary variable that equals one for cities with the ratio of FDI to GDP higher than the median of overall cities in current year, and zero otherwise. To test this hypothesis, we add the interaction terms of *hhi_bank*, *hhi_bank*^*2*^ and *highOI* into our baseline regression. The result in column (2) of Table 9 shows that the coefficient of the interaction term of *hhi_bank* and *highOI* is significantly negative, while the interaction term of *hhi_bank*^*2*^ and *highOI* is significantly positive. This suggests that the impact of bank market structure on firm innovation is more pronounced in cities with higher degree of openness to foreign markets than in those with lower degree of openness to foreign markets.

In summary, the results presented in this section contribute to the existing literature by highlighting that the non-linear relationship between bank market structure and firm innovation is particularly accentuated in cities with stronger IPR protection and a greater degree of openness to foreign markets when compared to their counterparts.

## 8. Conclusion and limitation

This paper examines the impact of bank market structure on firms’ innovation in an emerging market context, using China, the world’s largest emerging economy, as a case study. Our analysis is based on a sample of Chinese firms from 1998 to 2013. Our findings demonstrate a U-shaped relationship between bank market structure and firm innovation in China, with the financing channel playing a crucial role. We show that this relationship is mainly driven by small and innovative firms and it is more pronounced in regions with better IPR protection or higher degrees of openness to foreign markets.

This paper makes several contributions to the existing literature. First, unlike prior studies that focus on the linear relationship between bank market structure and firm innovation, our results confirm a U-shaped relationship where, as bank market structure shifts from concentrated to competitive, firm innovation initially decreases and subsequently increases. Second, we extend the literature by providing a detailed explanation of the underlying mechanisms and the features of the firms and cities that mainly drive the U-shaped relation. Finally, this study is one of the few investigations into the impact of bank market structure on firm innovation in emerging economies.

We acknowledge certain limitations in our study and identify potential avenues for future research. Our sample for the baseline regression is constrained by the availability of the data. However, we believe that this limitation does not significantly affect the non-linear relationship we’ve uncovered between bank market structure and firm innovation. Our data shows that after 2013, the bank market structure becomes more and more competitive, which indicates that as time goes on, more data present in the left-hand side of the U-shaped curve. Nevertheless, as long as we do not exclude the concentrated bank market structure from our dataset, we can still identify the non-linear relationship. To strengthen our hypothesis, we conducted additional analyses using data from listed companies, extending the sample period to 2019, and obtained consistent results. Nonetheless, we must acknowledge that if industrial data were available beyond 2013, the magnitudes of our estimates might differ. Furthermore, our study focused on China as a case study, given its status as the world’s largest emerging economy. Nevertheless, each country possesses a unique financial market and institutional structure. Future research could expand to cross-country comparisons and delve into how institutional structures influence the relationships between bank market structure and corporate innovation.

## References

[1] Solow R M. Technical change and the aggregate production function. The review of Economics and Statistics. 1957; 39: 312–320.

[2] Hsu P, Tian X, Xu Y. Financial Development and Innovation: Cross Country Evidence. Journal of Financial Economics. 2014; 112: 116–135.

[3] Ho C Y, Huang S, Shi H, Wu J. Financial deepening and innovation: The role of political institutions. World Development. 2018; 109;1–13.

[4] Moshirian F, Tian X, Zhang B, Zhang W. Stock market liberalization and innovation. Journal of Financial Economics. 2020; 139(3); 985–1014.

[5] Trinugroho I, Lao S H, Lee W C, Wiwoho J, Sergi B S. Effect of financial development on innovation: Roles of market institutions. Economic Modelling. 2021; 103; 105598.

[6] Wang X. Capital account liberalization, financial dependence and technological innovation: Cross-country evidence. Journal of Banking and Finance. 2022; 145; 106642.

[7] Carlin W, Mayer C. Finance, Investment, and Growth. Journal of Financial Economics. 2003; 69: 191–226.

[8] Benfratello L, Schiantarelli F, Sembenelli A. Banks and innovation: Microeconometric evidence on Italian firms. Journal of Financial Economics. 2008; 90: 197–217.

[9] Chava S, Oettl A, Subramanian A, Subramanian KV. Banking deregulation and innovation. Journal of Financial Economics. 2013; 109: 759–774.

[10] Cornaggia J, Mao Y, Tian X, Wolfe B. Does Banking Competition affect Innovation? Journal of Financial Economics. 2015; 115: 189–209.

[11] Guzman M. Bank structure, Capital Accumulation and Growth: a simple macroeconomic model. Economic Theory. 2000; 16: 421–455.

[12] Biswas S S, Koufopoulos K. Bank competition and financing efficiency under asymmetric information. Journal of corporate finance. 2020; 65: 101504.

[13] Petersen M A, Rajan R G. The Effect of Credit Market Competition on Lending Relationships. The Quarterly Journal of Economics. 1995; 110 (2): 407–443.

[14] Patti E B, Dell’Ariccia G. Bank Competitiona nd Firm Creation. Journal of Money, Credit and Banking. 2012; 36(2): 225–251.

[15] Huang H, Xu C. Institutions, Innovations, and Growth. American Economic Review 89. 1999; 438–443.

[16] Amore M, Schneider C, Zaldokas A. Credit Supply and Corporate Innovation. Journal of Financial Economics. 2013; 109: 835–855.

[17] Lind J T, Mehlum H. With or without U? The appropriate test for a U‐shaped relationship. Oxford bulletin of economics and statistics. 2010; 72(1): 109–118.

[18] Liu P, Li H. Does bank competition spur firm innovation? Journal of Applied Economics. 2020; 23(1); 519–538.

[19] Xin F, Zhang J, Guo Y, Liang S. Banking structure change and corporate innovation: evidence from Chinese city-branch data. Accounting & Finance. 2022; 62: 2057–2084.

[20] Xia Y, Liu P. The effects of bank competition on firm R&D investment: an inverted-U relationship. Chinese Management Studies. 2021; 15(3); 641–666.

[21] Zhang J, Zheng W, Xin F. Bank Deregulation, Structural Competition and Enterprises’ Innovation in China. China’s Industrial Economics. 2017; 10: 118–136.

[22] King R G, Levine R. Finance and growth: Schumpeter might be right. Quarterly Journal of Economics. 1993; 108: 717–737.

[23] Morales M. Financial intermediation in a model of growth through creative destruction. Macroeconomic Dynamics. 2003; 7(3): 363–393.

[24] Levine R. Finance and growth: theory and evidence. In: Aghion P., Durlauf S. (Eds.), Handbook of Economic Growth, vol. 1A., Elsevier, Amsterdam, Netherlands; 2005. pp. 865–934.

[25] Weinstein D E, Yafeh Y. On the costs of a bank-centered financial system: evidence from the changing main bank relations in Japan. Journal of Finance. 1998; 53: 635–672.

[26] Morck R, Nakamura M. Banks and corporate control in Japan. Journal of Finance. 1999; 54: 319–339.

[27] Shang H, Song Q, Wu Y. Credit market development and firm innovation: evidence from the People’s Republic of China. Journal of the Asia Pacific Economy. 2017; 22(1): 71–89.

[28] Riordan M H. Competition and bank performance: a theoretical perspective (No. 0026). 1992.

[29] Caminal R, Matutes C. Bank solvency, market structure, and monitoring incentives (No. 1665). CEPR Discussion Papers. 1997.

[30] Schnitzer M. Bank competition and enterprise restructuring in transition economies. Available at SSRN 147468. 1998.

[31] Schnitzer M. On the role of bank competition for corporate finance and corporate control in transition economies. Journal of Institutional and Theoretical Economics (JITE)/Zeitschrift für die gesamte Staatswissenschaft. 1999; 22–46.

[32] Ratti R A, Lee S, Seol Y. Bank concentration and financial constraints on firm-level investment in Europe. Journal of Banking and Finance. 2008; 32(12): 2684–2694.

[33] Smith R T. Banking competition and macroeconomic performance. Journal of Money, Credit and Banking. 1998; 793–815.

[34] Deng S, Mao C X, Xia C. Bank Geographic Diversification and Corporate Innovation: Evidence from the Lending Channel. Journal of Financial and Quantitative Analysis. 2021; 56(3): 1065–1096.

[35] Huang W, Wu Y, Deng L. Does banking competition stimulate regional innovation? Evidence from China. Pacific-Basin Finance Journal. 2021; 70: 101674.

[36] Xia Y, Liu P. Does Bank Competition Promote Corporate Green Innovation? Evidence from the Location of Bank Branches. China & World Economy. 2022; 30(2): 84–116.

[37] Tan X, Dong Y, Fang T. Bank Competition, Combination of Industry and Finance, and Enterprise Innovation: Evidence from China. Complexity. 2022.

[38] Li Y, Peng W. Bank price competition and enterprise innovation-Based on empirical evidence of Chinese A-share listed companies. International Review of Finance Analysis. 2024; 91; 103004.

[39] Chang T P, Hu J L, Chou R Y, Sun L. The sources of bank productivity growth in China during 2002–2009: A disaggregation view. Journal of Banking & Finance. 2012; 36: 1997–2006.

[40] Berger A N, Hasan I, Zhou M. Bank ownership and efficiency in China: What will happen in the world’s largest nation? Journal of Banking & Finance. 2009; 33: 113–130.

[41] Firth M, Lin C, Liu P, Wong M L. Inside the black box: Bank credit allocation in China’s private sector. Journal of Banking & Finance. 2009; 33: 1144–1155.

[42] Ferri G. Are New Tigers supplanting Old Mammoths in China’s banking system? Evidence from a sample of city commercial banks. Journal of Banking & Finance. 2009; 33: 131–140.

[43] Brandt L, Van Biesebroeck J, Zhang Y. Creative accounting or creative destruction? Firm-level productivity growth in Chinese manufacturing. Journal of Development Economics. 2012; 97: 339–351.

[44] Tan Y, Tian X, Zhang X, Zhao H. The real effect of partial privatization on corporate innovation: Evidence from China’s split share structure reform. Journal of Corporate Finance. 2020; 64: 101661.

[45] Chong T L, Lu L, Ongena S. Does banking competition alleviate or worsen credit constraints faced by small- and medium-sized enterprises? Evidence from China. Journal of Banking & Finance. 2013; 37: 3412–3424.

[46] Qian M, Yeung B Y. Bank financing and corporate governance. Journal of Corporate Finance. 2014; 32: 258–270.

[47] Jin G, Yu B, Shen K. Domestic trade and energy productivity in China: An inverted U-shaped relationship. Energy Economics. 2021; 97: 105234.

[48] Fan H, Lai E L C, Li Y A. Credit constraints, quality, and export prices: Theory and evidence from China. Journal of Comparative Economics. 2015; 43: 390–416.

[49] Manova K, Wei S, Zhang Z. Firm exports and multinational activity under credit constraints. Review of Economics and Statistics. 2015; 97: 574–588.

[50] Lai T, Qian Z, Wang L. WTO accession, foreign bank entry, and the productivity of Chinese manufacturing firms. Journal of Comparative Economics. 2016; 44: 326–342.

[51] Dong B, Guo Y, Hu X. Intellectual property rights protection and export product quality: Evidence from China. International Review of Economics and Finance. 2022; 77: 143–158. doi: 10.1016/j.iref.2021.09.006

[52] Fan G, Wang X L, Zhu H P. NERI INDEX of marketization of China’s provinces 2011 report. Beijing (in Chinese): Economics Science Press (Beijing: Jing ji ke xue chu ban she); 2011.

[53] Wang X, Fan G, Yu J. Marketization index of China’s provinces: NERI report 2016. Beijing (in Chinese): Social Science Academic Press (Beijing: She hui ke xue wen xian chu ban she); 2016.

[54] Chen C, Chang L, Zhang Y. The role of foreign direct investment in China’s post-1978 economic development. World Development. 1995; 23(4): 691–703. doi: 10.1016/0305-750X(94)00143-M

[55] Jiang H, Liang Y, Pan S. Foreign direct investment and regional innovation: Evidence from China. World Economy. 2021; 45: 1876–1909. doi: 10.1111/twec.13205

